# Volatile Organic Compounds from an Indoor Pest, *Luprops tristis*, Collected by a Novel Glass Chamber and Their Implications for Human Health

**DOI:** 10.3390/insects17060617

**Published:** 2026-06-11

**Authors:** Sajidha Mohammed, K. S. Shameer, Thomas Hesselberg, K. U. M. A. Rafeeq

**Affiliations:** 1 Department of Zoology, MES Mampad College (Autonomous), University of Calicut, Malappuram 676542, India; sajidha@mesmampadcollege.edu.in; 2 Department of Agricultural Sciences, University of Helsinki, P.O. Box 27, FI-00014 Helsinki, Finland; shameer.ks@helsinki.fi; 3 Department for Continuing Education, University of Oxford, Oxford OX1 2JA, UK; 4 Department of Biology, University of Oxford, Oxford OX1 3EL, UK

**Keywords:** indoor insect pests, *Luprops tristis*, insect volatiles, aggregation, glass chamber, allergies

## Abstract

Insects secrete volatile organic compounds (VOCs) for various reasons, such as intra- or inter-species communication, attracting mates, or repelling predators. The volatiles from indoor insect pests, e.g., phenolic secretions, can impact inhabitants in various ways, causing allergies, skin and eye irritations, etc. The Mupli beetle (*Luprops tristis* Fabricius, 1801) is one such nuisance pest that aggregates in great numbers in indoor spaces, especially near rubber plantations in tropical African and Asian countries. This study aimed to understand the whole-body volatilome of *L. tristis*, comprising the first detailed study of volatiles in this insect, particularly under aggregation and laboratory conditions. Whole-body VOCs were collected from sets of 500 and 1000 beetles at different time intervals and analysed by solvent-assisted desorption followed by gas chromatography–mass spectrometry (GC-MS). Compounds released by the Mupli beetle, such as 1-Octadecanesulphonyl chloride, Decane-1,1′-oxybis-, n-Nonadecanol-1 and n-Heptadecanol-1, are reported in the literature to be allergens that cause allergic reactions such as skin and eye irritations in humans. This understanding may indicate the possible reasons for the allergic reactions in people living in these insect-inhabited indoor spaces. We also report and describe the design and development of an economically feasible glass chamber for the dynamic headspace collection of volatiles released by these beetles.

## Simple Summary

Some insects that live close to humans release chemicals into the air, which may affect the health of people living nearby. The Mupli beetle is a common indoor pest in areas close to rubber plantations in India, where it enters houses in very large numbers and is reported to cause skin and eye irritation in humans. This study aimed to identify the chemical substances released by these beetles to explore whether they could be linked to allergic and respiratory issues in humans. We collected the airborne chemicals released by groups of beetles under controlled conditions and identified them using a chemical analysis that separates and detects different substances. Several of the chemicals detected were compared with chemicals from previous studies and found to cause allergic reactions or irritation in people. These findings help explain why people living in beetle-infested houses may experience health problems. In addition, we designed a simple and low-cost glass chamber to collect insect-released chemicals, which can be used for studying other insects as well. This work is valuable to society because it introduces a new method of collecting airborne chemicals released by insects, adds to our understanding of how indoor insects can affect human health and supports better pest management and healthier living environments.

## 1. Introduction

Insects communicate using a cocktail of volatile organic compounds (VOCs), the study of which can provide valuable insight into their chemical nature and impact in various contexts, particularly concerning insect allergies caused by indoor insect pests [1]. Insect allergies, which typically occur around the areas of insect bites or stings, result in symptoms such as pain, itching, swelling, and redness. Airborne particles from cockroaches, cat fleas, and cloth moths are a significant health concern for individuals in ethnically, economically, and socially marginalised groups, who exhibit a greater number of positive reactions to allergens [2,3]. Studies focusing on allergies from house dust mites [4] have shown that they can cause asthma, allergic rhinitis or both. Similarly, occupational allergies [5] can occur when workers are exposed to a higher density of insects such as the larvae of flies and moths [6], crickets and locusts [7,8], Mediterranean flour moths (*Ephestia kuehniella*) [9], fruit flies [10] and cockroaches [11]. Such allergies include rhinitis, asthma, and immediate systemic allergic reactions.

Most insect volatiles function as semiochemicals that cause behavioural or physiological responses in other individuals of the same or different species [12,13]. These chemical compounds are usually between 5 and 20 atoms of carbon with molecular weights ranging from 80 to 300 Daltons and include hydrocarbons, acetate esters, alcohols, acids, epoxides, ketones, isoprenoids, and triacyl glyceride groups [14,15]. The volatile chemical signals used for insect communication include intraspecific pheromones and interspecific allelochemicals, with some of them acting as both pheromones and allelochemical signals [16]. The qualitative and quantitative assessments of these multicomponent semiochemicals conducted to study the negative impacts of the insects can be effectively carried out using various headspace and air entrainment techniques [17].

Headspace analysis, where a volume of vapour or gas is collected above a study sample in an enclosed space, has always been a crucial part of volatile studies [18]. The complications of analysing such volatiles from insects are made much more difficult by their small size and active nature. Since the volatiles are found in very small amounts, analysis of them requires the development of very sensitive techniques [18].

Previous studies have used a range of different techniques; for example, a study used 30 L glass jars with compressed air filtered and further drawn at a rate of 300 mL min^−1^ through a stainless-steel cartridge filled with an adsorbent to study caterpillar-induced large-sized plant volatiles [19]. Another used Erlenmeyer flasks to study faeces of *T. confusum* host larvae by using charcoal-filtered air at 160 mL/min and tenax TA to adsorb them at room temperature [20], and one study used a clear I-Chem jar (150 mL, 6 cm wide by 7 cm high, VWR LLC, Radnor, PA, USA) with an air at rate of 0.2 L/min as a carrier gas to release aphrodisiac pheromones of small cabbage white butterflies [21]. Similarly, a 2 L round-bottom flask fitted to a gas-washing piece with the inlet coupled with an activated-charcoal-filled glass tube filled with 250 mg of preconditioned type Q, 80–100 mesh Porapak (Sigma-Aldrich, St. Louis, MO, USA) and connected to a vacuum pump to provide an airflow of approximately 3 L/h was used to study the sex pheromone of the mealybug *Dysmicoccus grassii* Leonardi [22], and two 3L glass chambers, with purified air injected at a rate of 4 L/m and a solid-phase adsorbent trap containing Super-Q (80/100 mesh; Alltech, Deerfield, IL, USA), were used to study the alarm pheromone of winged dispersal morphs of aphids [23]. One of the most efficient techniques is trapping the headspace volatiles for specific periods onto a sorbent material, which is then subjected to chemical analysis. Dynamic headspace sampling (involving air flow at a particular pressure) helps direct the concentration of semiochemicals readily released from insects onto the adsorbent tubes [24].

The mupli beetle (*Luprops tristis*) is a nuisance pest that aggregates in millions in indoor spaces near rubber plantations. Its larval instars, pupae and teneral adults feed on tender rubber leaves high in nitrogen and moisture [25,26]. The life cycle of this species is closely synchronised with climatic conditions, with beetles emerging from an 11-month diapause at the onset of rain [27]. Following this, they migrate from moist plantation floors containing rubber leaf litter to comparatively drier indoor environments. These beetles are particularly attracted to tile-roofed and palm-frond thatched indoor environments, which retain higher humidity than concrete structures [27]. Eventually, these approximately 8 mm long insects fall from the roof onto food items and bed spaces, creating a visual disturbance [28]. Mupli beetles produce phenolic secretions when disturbed and are known to cause skin and eye irritations [29]. Inhalant indirect allergies such as colds, a cough, and sneezing can also arise from exposure to these beetles, in addition to the more direct allergic reactions such as skin irritations and skin burns [30]. We hypothesise that the semiochemicals released by this indoor species may be the reason for allergies in inhabitants. Hence, this study was conducted to identify the volatile organic compounds released by these insects and find out whether they might have any hazardous effects on human beings. For the collection of volatiles released by *L. tristis*, an economically feasible glass chamber was designed and fabricated. In this paper, we also describe this new device and its use in the collection of volatiles from Mupli beetles for dynamic headspace analysis.

## 2. Materials and Methods

### 2.1. Design and Fabrication of the Glass Chamber

To facilitate the profiling of the whole-body volatilome of *Luprops tristis*, an economically feasible glass chamber for headspace collection of volatiles from the insects under laboratory conditions was designed and fabricated.

### 2.2. Collection of Insects from Sampling Sites for Direct Semiochemical Analysis

The Mupli beetles were collected from a rural house for 2 months (April–May 2022) in Malappuram (Coordinates: 11° N 76° E), a district in the South Indian state of Kerala. Collection methods such as hand-picking and brushing were used to collect the insects in a glass jar from the sampling site. The lid of the glass jar was perforated to allow the required amount of gas exchange.

### 2.3. Dynamic Headspace Analysis of Volatiles

The collected insects were transferred into a headspace volatile collection chamber maintained at room temperature. Air at a particular flow rate (1000 mL/m) was allowed to pass through the silicon tubes to activated charcoal (200 mg, 40–80 mesh size) embedded in a glass tube to remove contaminants. The filtered air then entered the inlet of the chamber through a silicon tube. The volatiles that were released by the beetles and had passed through the perforations in the glass disc were collected at the headspace portion of the glass chamber and then pushed, along with the filtered air, to the outlet into a different glass tube containing activated charcoal (200 mg, 40–80 mesh size), acting as adsorbent material. Whole-body VOCs were analysed from freshly collected groups of 500 and 1000 beetles at different time intervals (12, 24, 48, and 72 h), corresponding to insect densities of 0.5 and 1 beetle/mL, respectively. The activated charcoal was subjected to solvent-assisted desorption using n-hexane (Emplura-Merck, Darmstadt, Germany) for 24 h and analysed using gas chromatography–mass-mass spectrometry (GC-MS) to characterise the compounds present in the whole-body headspace experiment. A control experiment replicating the same procedure but devoid of the beetles was also conducted for each period.

### 2.4. Gas Chromatography–Mass Spectrometry Specifications

A Shimadzu GC-MS with the model number QP2010S was used for the volatile analysis. The column used was ELITE-5MS with 30 m length, 0.25 mm internal diameter and 0.25 µm thickness. The Software used was GCMS Solutions (v. QP2010) with the library NIST 11 and WILEY 8.

During the analysis, the Column Oven Temperature was maintained at 70.0 °C, and the Injection Temperature was set to 250.00 °C. The mode of injection was splitless sampling. Time was set to 2.00 min, and the Flow Control Mode was Linear Velocity. The pressure was adjusted to 61.5 kPa, the total flow was set to 54.0 mL/min, the column flow was set to 1.00 mL/min, the Linear Velocity was set to 36.7 cm/sec, the Purge Flow was set to 3.0 mL/min and the split ratio was set to 50.0.

The Ion Source Temperature was maintained at 200.00 °C, the Interface Temperature was kept at 280.00 °C and the Solvent Cut Time was kept at 4.00 min. The Detector Gain Mode was relative Detector Gain at 1.12 kV + 0.20 kV and threshold at 1000. The start time was 4.10 min, and end time was 35.75 min. The ACQ Mode was scanning with an event time of 0.30 s and a scan speed of 1666.

### 2.5. Statistical Analysis

The data was analysed using R Studio (version 4.0.5) [31] with the tidyverse and ggplot 2 packages. To assess the differences in the release of compounds over time, a one-way ANOVA was used, with number of compounds and time serving as fixed factors. Graphical visualisations were generated using ggplot 2. Statistical significance was set at *p* < 0.05.

## 3. Results

### 3.1. Design and Fabrication of the Glass Chamber

**Figure 1:**
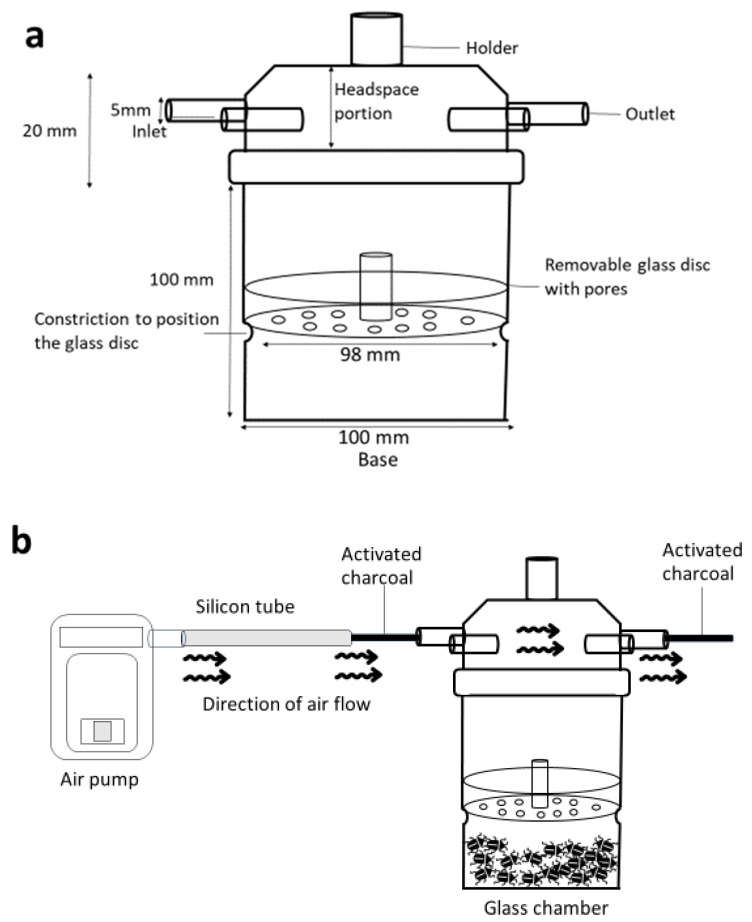
(**a**) The glass chamber designed to collect the volatilome of *L. tristis*. (**b**) The experimental setup. Air was pumped through activated charcoal into the inlet, which collected the air containing volatiles from beetles and pushed these volatiles into the activated charcoal tube attached to the outlet, acting as adsorbent.

The glass chamber was designed to have a total volume of 1000 mL (Figure 1). It has two inlets and two outlets, each on the glass lid, and a slight constriction on the base of the chamber to allow a removable glass disc to rest on it to prevent the insects placed at the bottom of the chamber from moving upwards. The headspace portion is a removable glass lid with a thickness of 0.5 mm, a diameter of 100 mm, a height of 50 mm and an approximate capacity of 200 mL. It acts as an entry space with which to position the insects inside the insect chamber. This cover has a fattened edge similar to the top edge of the insect chamber and is heavy enough to block passage the of air into or outside of the insect housing chamber. The headspace portion has a knob made of glass, measuring 50 mm in length and 25 mm in diameter, with a constriction to hold the portion better. The inlets and outlets are 25 mm in length and 5 mm in diameter. The filtered air enters the chamber through the inlet and leaves through the two outlets, carrying the insect volatilome from the chamber through the outlets to the adsorbent material embedded in the collection tube.

The base of the chamber that houses the insects has a volume of approximately 800 mL. A glass disc with a knob, with a 98 mm diameter, a thickness of 0.5 mm, and a height of 15 mm, was placed at the constriction near the base of the chamber to restrict the insects at the bottom of the chamber from moving towards the inlets and outlets, thus blocking the pathway of the air passage. This separation glass disc has 10 perforations, each measuring 2 mm in diameter to allow movement of air between the bottom and upper parts.

### 3.2. Dynamic Headspace Analysis of Volatiles

The GC-MS profiles of the candidate compounds from *L. tristis* in the glass chamber with 500 and 1000 individuals showed hydrocarbons, alcohols, esters, aldehydes and ethers. Forty-one volatile compounds were recorded, of which 18 have known functions and 23 have unknown functions (Table 1 and Table 2). Among the compounds with known functions, eleven are reported to cause hazardous effects such as allergies in human beings (Table 3). The compounds were recorded along with their retention time (the time taken to pass through the chromatography column and elute at the detector), peak area percentage (the area under the peak of a specific compound as a percentage of the total area of all peaks present) and frequency (the number of times a compound appeared during the experiment, with different numbers of beetles and during different periods).

Among the hydrocarbons, four of them were identified as cuticular hydrocarbons, five were defensive compounds, and three were attractants (Table 1). Amongst these, the most common and abundant compound detected in the whole-body assay was 2-methyloctacosane. The retention time of the compounds identified was between 10 to 31 min. Four alcohols were identified (Table 1), among which two are defensive compounds, and the remaining two are sex pheromones. All the compounds detected in the whole-body assay were detected only once. The retention time of the compounds identified was between 18 and 25 min. The function of the only aldehyde compound (Table 1) detected was to serve as a sex pheromone, and the compound was only detected once, with the retention time being 16 min. The ester compound detected was found to have a defensive function (Table 1), which was only observed once, with a retention time of 24 min.

Among the twelve hydrocarbons identified, the most common and abundant compound detected in the whole-body assay was Heptadecane, 2,6,10,15-tetramethyl- (Table 2). The retention times of the compounds detected varied between 18 and 33 min.

Three alcohol compounds were detected only once, with retention times between 12 and 23 min (Table 2). Seven esters were detected in the whole-body assay only once, with their retention times between 21 and 28 min (Table 2). The ester compound with the unknown function was detected only once, with its retention time being 16 min (Table 2).

**Figure 2:**
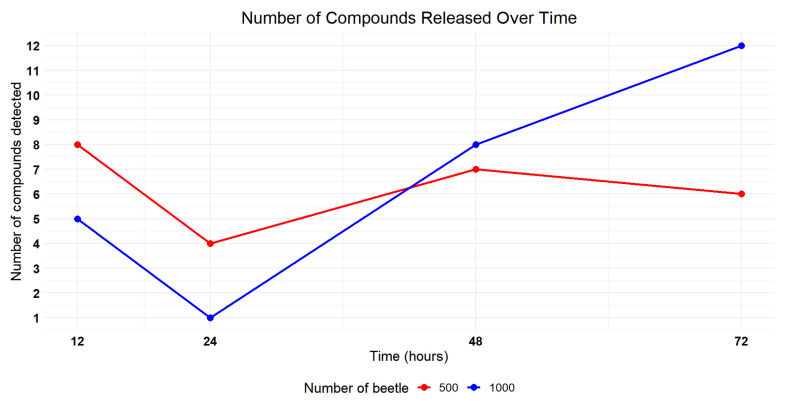
Number of compounds detected in the GC-MS analysis of headspace volatiles with 500 and 1000 *L. tristis* beetles at different timepoints. A control without the beetles was also maintained for every time period.

The number of compounds detected during the headspace volatile analysis with 500 and 1000 beetles for different time periods, namely, 12, 24, 48 and 72 h, showed variations (Figure 2).

However, two-way ANOVA revealed that neither time (df = 3, F = 1.694, *p* = 0.338) nor beetle density (df = 1, F = 0.014, *p* = 0.914) had a statistically significant effect on the number of compounds detected.

Although descriptive trends were observed, such as relatively higher compound detection at 12 h, a decline at 24 h, and a subsequent increase at 48 h, these changes were not statistically significant. Similarly, while the 1000-beetle treatment appeared to yield slightly higher compound numbers at later timepoints (particularly at 48 and 72 h), this apparent increase was not statistically significant.

Overall, the results indicate that neither exposure duration nor beetle density significantly influenced the number of volatile compounds detected under the conditions tested.

## 4. Discussion

The collection of insect volatile organic compounds is preferably done in glass materials to avoid the presence of contaminants, such as plastics. The current invention, which utilises a glass chamber, was designed to eliminate rubber joints, which can be an issue when using other collection methods such as modified Erlenmeyer flasks for studying insects [20,22]. Our glass chamber, therefore, allows more effective collection of volatiles by reducing contamination from unwanted compounds usually occurring due to the chemical nature of the joined parts. This portable system can also be directly connected to a GC-MS system for real-time volatile analysis. Due to its compact size, the volatiles released by the insect tend to get concentrated in a smaller area. Moreover, the amount of air to be pushed and pulled through it is comparatively smaller, also increasing the chance of trapping more compounds. Any insects larger than the holes in the separation disk may be placed inside the chamber, as this prevents them from moving upwards and blocking the inlets and outlets. Moreover, reducing the dimensions of the separation disc allows the study of much smaller insects.

This study aimed at describing the volatiles released by the Mupli beetle (*Luprops tristis*) in indoor spaces using the specifically designed headspace glass chamber, which facilitated standardised and quantitative analytical profiling of the insect’s whole-body volatilome. During the whole-body headspace collection, using this glass chamber, we detected and identified a range of compounds belonging to different classes. Analysis of the volatiles adsorbed onto activated charcoal released by the Mupli beetles placed in the glass chamber revealed 41 different compounds with retention times between 10 and 31 min. This is similar to an analysis done on defensive gland secretions of the adult beetle [51], which also identified 29 compounds, three of which were the same compounds found in our study (decane, hepatadecane and tetradecane). Out of the 41 compounds found in this study, 18 were identified as known pheromones based on previous work on various other insects (see Table 1 and Table 2). These included eight defensive pheromones, six sex pheromones and four cuticular hydrocarbons. Whereas among the unidentified chemical volatiles, there were 12 hydrocarbons, 3 alcohols, 7 esters and 1 ether compound. Notably, no existing research supports their presence as insect-emitted volatiles, suggesting the possibility of novel or previously unreported compounds.

*Luprops tristis* is a nocturnal insect, and each set of experiments in this study was initiated during its peak activity period in the evening. As a result, the 12 h experiment recorded a higher number of volatiles, as the entire sampling period coincided with the beetles’ most active phase (Figure 2). In contrast, the 24 h experiment showed a reduction in the total volatiles detected, as the latter half of the sampling period coincided with their inactive daytime phase lasting approximately 12 h. Moreover, the volatile nature of the compounds increased the likelihood of losses from the first 12 h during the extended collection period. The experiments exceeding 24 h encompassed more than one day–night cycle of the beetle. Consequently, a slightly higher number of volatile compounds were obtained when a greater number of beetles were placed in the glass chamber for longer durations. The present experiment, which was conducted from April to May, recorded data mostly during the active season of the Mupli beetle, whose large-scale seasonal aggregations in every generation occur after 9 months of an inactive dormancy phase [29]. Their aggregations after the summer showers, usually during the end of March, usually occur in indoor spaces near rubber plantation sites, which allows them to feed on tender rubber leaves high in nitrogen and moisture [52].

Although the semiochemical systems of insects are considered to be of potential importance for intra-specific communication [53], such chemicals might also form a part of their irritant secretions. The presence of insects has at times been related to the appearance of certain allergies [54]. The Mupli beetle has been proven to cause allergies via its volatile secretions [52], sometimes also resulting in keratoconjunctivitis [55]. The probable reason these beetles have such an influence lies in their huge aggregations. To a lesser extent, the same may be the case with several other insects. Interestingly, 11 of the 41 compounds detected during the assay were classified as hazardous chemicals (Table 3). These included six hydrocarbons, four alcohols and one ester compound. The nature of their negative effects on human health is potentially manifold but mostly included respiratory impacts.

Inhalant allergies from such allergens are mostly concentrated in the vicinity of the insects and have been observed during the metamorphosis of a huge density of chironomid midges in Japan [56], which usually results in asthma [57,58]. A similar case of inhalant allergy was also observed to be caused by ladybird beetles, although they are generally considered a pest control option for aphids and scale insects [1]. Ladybird beetles can also show a seasonal appearance similar to that of the Mupli beetle, although this is during winter. They have been observed to cause asthma, allergic rhinitis and angioedema, but this is due to the presence of proteins such as Har a 1 and Har a 2 [59,60,61]. Similar allergies arising from cockroaches were found to affect inner-city children exposed to 12 identified allergens from cockroach exoskeletons and faeces [62,63].

As the large-scale colonisation of these insects in indoor spaces in India, particularly houses, is unavoidable during certain seasons of the year, the hazardous compounds that are secreted by these insects need to be better understood. The suspected harmful compounds include Heneicosane, a hydrocarbon identified during our assay, which is a skin and eye irritant [50], and 1-Octadecanesulphonyl chloride, which might show corrosive effects [50]. Similarly, Heptadecane and Eicosane are hydrocarbons that are known to cause respiration hazards [50], while another hydrocarbon, Dodecane, 1-fluoro-, is known to cause Acute Oral Toxicity [50], and Decane, 1,1′-oxybis-, n-Nonadecanol-1 and n-Heptadecanol-1 are both irritants [50]. Finally, the alcohol compound 1-Hexanol, 5-methyl-2-(1-methylethyl)- causes acute allergic effects, and 1-Heneicosanol might be hazardous to the aquatic environment [50]. The only ester compound that is hazardous among the ones detected is sulphurous acid, decyl 2-propyl ester, and it causes long-term hazards in the aquatic environment [50]. However, detailed studies on the chemical nature of the compounds identified in this study are required to understand the effects on humans and their roles in inflicting asthma and other allergies.

## 5. Conclusions

Insects have always been considered a notable cause of inhalant allergies, especially in indoor spaces, depending on the different environmental conditions and the kinds of patients. This study has shown that the Mupli beetle releases certain hazardous compounds that may contribute to health issues among human inhabitants of indoor spaces where huge aggregations of this insect occur. Studying the extent of the allergenicity, particularly of the most common volatiles, using immunoglobulin and skin prick tests can further aid in the development of better treatments. The elucidation of the influences of these small but ubiquitous animals on indoor air quality is a matter of serious concern.

## Figures and Tables

**Table 1:** Volatile organic compounds released by *L. tristis* and their functions already reported in the literature. The compounds are arranged according to classes, such as hydrocarbons, alcohols, aldehydes and esters.

Retention Time (min)	Compound	Class of Compound	Area (%)	Frequency	Function
10.43	Nonadecane, 2-methyl-	Hydrocarbon	19.17	1	Cuticular Hydrocarbon [32]
21.320	Eicosane	Hydrocarbon	3.57	2	Defensive [33]
23.473	Heptadecane	Hydrocarbon	9.94	1	Defensive [34]
24.515	Octane, 3,4,5,6-tetramethyl-	Hydrocarbon	2.05	1	Defensive [35]
24.520	Eicosane, 10-methyl-	Hydrocarbon	13.89	1	Sex Pheromone [36]
25.740	2-methyloctacosane	Hydrocarbon	14.76	3	Cuticular Hydrocarbon [37]
27.024	Eicosane, 2-methyl-	Hydrocarbon	10.57	2	Sex Pheromone [38]
27.031	Heptadecane, 9-octyl-	Hydrocarbon	4.28	2	Defensive [39,40]
27.067	Tritetracontane	Hydrocarbon	11.05	2	Cuticular Hydrocarbon [41,42]
27.087	Heneicosane	Hydrocarbon	13.48	2	Sex Pheromone [43]
28.465	Tetracosane, 11-decyl-	Hydrocarbon	11.41	1	Cuticular Hydrocarbon [39]
31.320	Undecane, 2,7-dimethyl	Hydrocarbon	5.76	1	Defensive [34]
18.969	1-Heneicosanol	Alcohol	5.71	1	Defensive [44]
22.453	1-Hexanol, 5-methyl-2-(1-methylethyl)-	Alcohol	1.36	1	Defensive Allomone [45]
24.531	2-Nonen-1-ol, (E)	Alcohol	2.01	1	Sex Pheromone [46]
25.627	n-Heptadecanol-1-	Alcohol	3.11	1	Sex Pheromone [47]
16.449	E-15-Heptadecenal	Aldehyde	2.67	1	Sex Pheromone [48]
24.510	Sulfurous acid, 2-propyl tridecyl ester	Ester	9.99	1	Defensive Pheromone [49]

**Table 2:** Volatile organic compounds released by *L. tristis* with unknown functions. The compounds are arranged according to class as hydrocarbon, alcohol, ester and ether, respectively.

Retention Time (min)	Compound	Class of Compound	Area (%)	Frequency
18.685	1,3-Dioxolane, 2-(2-propenyl)-	Hydrocarbon	1.01	1
19.656	2-Thiophenepropanamine, N, N-dimethyl-	Hydrocarbon	13.27	1
23.490	Di-n-decylsulfone	Hydrocarbon	1.49	1
25.724	Heptadecane, 2,6,10,15-tetramethyl-	Hydrocarbon	11.38	2
29.848	2-Bromotetradecane	Hydrocarbon	9.01	1
29.880	Decane, 1,1′-oxybis-	Hydrocarbon	4	1
31.341	Dodecane, 1-fluoro-	Hydrocarbon	4.46	1
31.357	Nonadecane, 1-bromo-	Hydrocarbon	8.87	1
32.130	1,1,3,3-Tetraallyl-1,3-disilacyclobutane	Hydrocarbon	4.35	1
32.540	2-Methyl-5-t-butyl-1,3-oxathiane	Hydrocarbon	4.29	1
33.071	1-Octadecanesulphonyl chloride	Hydrocarbon	6.62	1
33.631	2,3-O-Benzal-d-mannosan	Hydrocarbon	8.05	1
12.607	Phenol, 2,4-bis(1,1-dimethylethyl)-	Alcohol	10.18	1
21.370	Melochinin	Alcohol	2.06	1
23.384	n-Nonadecanol-1	Alcohol	6.63	1
21.268	Trifluoroacetic acid, pentadecyl ester	Ester	7.73	1
22.394	Oxalic acid, allyl decyl ester	Ester	2.33	1
22.443	Oxalic acid, decyl propyl ester	Ester	6.12	1
25.695	Oxalic acid, 6-ethyloct-3-yl heptyl ester	Ester	2.94	1
27.070	Stearic acid, 3-(octadecyloxy)propyl ester	Ester	3.27	1
28.427	Methoxyacetic acid, 3-tetradecyl ester	Ester	10.43	1
28.436	Sulfurous acid, decyl 2-propyl ester	Ester	3.62	1
16.770	1-(2-Methoxyethoxy)-2-methyl-2-propanol, methyl ether	Ether	4.82	1

**Table 3:** Volatile organic compounds released by *L. tristis* and identified as hazardous compounds.

Nature	Compound	Effect
Hydrocarbon	Heneicosane	Skin & Eye Irritations [50]
Hydrocarbon	1-Octadecanesulphonyl chloride	Skin corrosion/irritation [50]
Hydrocarbon	Heptadecane	Aspiration hazard [50]
Hydrocarbon	Eicosane	Aspiration hazard [50]
Alcohol	1-Heneicosanol	Slight Hazard to aquatic environment [50]
Ester	Sulfurous acid, decyl 2-propyl ester	Long term Hazard to aquatic environment [50]
Hydrocarbon	Dodecane, 1-fluoro-	Acute Oral Toxicity [50]
Alcohol	1-Hexanol, 5-methyl-2-(1-methylethyl)-	Acute Allergic Effects [50]
Hydrocarbon	Decane, 1,1′-oxybis-	Irritant [50]
Alcohol	n-Nonadecanol-1	Irritant [50]
Alcohol	n-Heptadecanol-1	Irritant [50]

## Data Availability

The original contributions presented in this study are included in the article. Further inquiries can be directed to the corresponding authors.
